# Finger-Equipped Electrode Electrical Stimulation for Severe Upper-Limb Paresis in the Acute Phase of Stroke: A Retrospective Case Series

**DOI:** 10.7759/cureus.93838

**Published:** 2025-10-04

**Authors:** Takashi Nakamori, Shota Kitayama, Kei Hamamachi

**Affiliations:** 1 Department of Occupational Therapy, Faculty of Health Sciences, Kansai University of Health Sciences, Osaka, JPN; 2 Department of Rehabilitation, Okanami General Hospital, Mie, JPN

**Keywords:** acute-phase stroke, electrical stimulation, fugl-meyer assessment, neuroplasticity, upper-limb paresis

## Abstract

Aim and objective

Severe upper-limb paresis during the acute phase of stroke significantly limits functional recovery. Finger-equipped electrode electrical stimulation (FEE-ES) enables therapists to synchronize electrical stimulation (ES) with the patient’s motor intent, even in the absence of voluntary movement. However, evidence regarding its feasibility and preliminary therapeutic effects in the acute phase of stroke remains limited. Therefore, this study aimed to evaluate the feasibility, safety, and potential therapeutic effects of FEE-ES in patients in the acute phase of stroke who present with severe upper-limb paresis.

Materials and methods

We retrospectively examined six patients (mean age: 56.0 ± 15.0 years) with a first-ever stroke and severe upper-limb paresis (baseline Fugl-Meyer Assessment for Upper Extremity (FMA-UE) ≤19), without severe cognitive or attentional deficits. FEE-ES was applied for approximately one month during the acute phase as part of the rehabilitation program. Upper-limb motor function was assessed using the FMA-UE and the upper-limb items of the Stroke Impairment Assessment Set (SIAS). Spasticity and skin integrity were monitored throughout the intervention.

Results

The median baseline FMA-UE score was 4.0 (range: 4-6) points, which improved to 16.0 (range: 7-27) points after one month (p = 0.031, Wilcoxon signed-rank exact test; effect size r = 0.86). The Hodges-Lehmann estimator indicated a median improvement of 11 points (95% confidence interval: 3-23). Three patients exceeded the minimal clinically important difference (MCID) of 10 points. The median SIAS-Knee-Mouth and Finger Function scores increased from 0 to 1.5, and from 0.5 to 1.0, respectively. However, no increase in spasticity or skin complications was observed. Four participants had follow-up assessments at two months after stroke onset, and all exhibited continued functional gains.

Conclusion

This retrospective case series demonstrated that FEE-ES in the acute phase of stroke is feasible, safe, and may be associated with significant improvements in upper-limb motor function in patients with severe paresis. Notably, some individuals exhibited continued functional gains beyond the acute stage, suggesting that early synchronization of motor intent with ES may promote long-term recovery. Although preliminary, these findings support the potential of FEE-ES as an adjunct rehabilitation strategy and highlight the need for additional controlled studies to confirm efficacy and optimize protocols.

## Introduction

Upper-limb motor paresis occurs in approximately 70% of patients following cerebrovascular disorders, and only approximately 20% of those with severe paresis achieve complete functional recovery [1]. Impaired upper-limb function significantly limits independence in activities of daily living (ADLs) and reduces quality of life (QOL), highlighting the urgent need for effective rehabilitation interventions. Electrical stimulation (ES) therapy is one such promising approach. Among its modalities, electromyography (EMG)-triggered neuromuscular electrical stimulation (NMES), which synchronizes ES with voluntary muscle activity, has demonstrated efficacy in improving motor function [2]. However, detecting voluntary movements in patients with severe paresis is often difficult, limiting the applicability of EMG-triggered stimulation. To address this challenge, a finger-equipped electrode electrical stimulation (FEE-ES) has been developed as a novel therapeutic approach. Unlike conventional EMG-triggered NMES, FEE-ES enables therapists to deliver stimuli manually through finger-attached electrodes, allowing ES to be synchronized with the patient’s motor intention, even when voluntary EMG activity is undetectable [3]. This key technical difference broadens the potential application of ES to patients with severe upper-limb paresis who would otherwise be excluded from EMG-triggered therapies.

While FEE-ES has demonstrated clinical utility in chronic stroke [3], its application in the acute phase remains underexplored, with evidence limited to case reports and lacking systematic evaluation [4]. Given that the acute phase of stroke represents a critical period for neuroplasticity and functional recovery [5], introducing FEE-ES during this stage could offer substantial benefits. However, few studies have examined its feasibility, safety, and preliminary effects in patients with acute-phase stroke and severe upper-limb paresis, underscoring the need for empirical data.

Therefore, the present study retrospectively analyzed the clinical course of six such patients who underwent FEE-ES in the acute phase to evaluate its feasibility, safety, and preliminary therapeutic effects.

## Materials and methods

Participants

This study included patients who experienced their first-ever stroke between September 2020 and March 2024, presented with severe upper-limb paresis (Fugl-Meyer Assessment for Upper Extremity (FMA-UE) score ≤19 points at baseline) [6,7], and underwent FEE-ES during the acute phase for approximately one month.

The clinical criteria for FEE-ES administration were as follows: (1) no significant impairment of consciousness (Japan Coma Scale score I or lower) [8], and (2) absence of attention deficits or hemispatial neglect that could interfere with safe and effective use. Patients with mild-to-moderate paresis (FMA-UE >19), recurrent stroke, severe cognitive impairment, contraindications for ES, or early discontinuation of FEE-ES before completing the planned one-month intervention were excluded. During the study period, several inpatients with acute stroke received FEE-ES; however, only six patients met the inclusion criteria and were included in the analysis (Cases 1-6; Table 1). Given the retrospective nature of this study, the total number of screened and excluded patients could not be reliably determined from historical records.

**Table 1:** Characteristics of the study participants Aff. Hemi., affected hemisphere; CH, cerebral hemorrhage; CI, cerebral infarction; F, female; FF, finger function test; FMA-UE, Fugl-Meyer assessment for upper extremity; KM, knee-mouth test; Lt, left; M, male; MAS, modified Ashworth scale; Rt, right; SD, standard deviation; SIAS-M, stroke impairment assessment set-motor

Case	Sex	Age	Diagnosis	Aff hemi.	Lesion site	Surgery	Days from onset	FMA-UE	SIAS-M		MAS
									KM	FF	
1	F	72	CH	Lt	Putamen	Craniotomy with hematoma removal	6	4	0	0	0
2	M	47	CH	Rt	Putamen	Conservative	3	4	0	0	0
3	M	69	CI	Rt	Internal capsule	Conservative	7	4	1	1	1
4	M	70	CI	Lt	Pons	Conservative	1	4	0	0	0
5	M	45	CI	Rt	Putamen	Mechanical thrombectomy	6	4	1	1	1
6	M	33	CH	Lt	Putamen	Conservative	4	6	0	1	0
Mean (SD)		56.0 (15.0)					4.5 (2.1)				

The mean age of the included patients was 56.0 ± 15.0 years. All patients initiated occupational therapy within one week of stroke onset (mean: 4.5 ± 2.1 days). Baseline FMA-UE scores were 4 points in Cases 1-5 and 6 points in Case 6. In the upper-limb motor items of the Stroke Impairment Assessment Set (SIAS) [9], all patients scored ≤1 point on both the Knee-Mouth and Finger Function tests, indicating severe paresis. Muscle tone of the affected upper limb was also evaluated using the Modified Ashworth Scale (MAS) [10], with all patients scoring 0 or 1, indicating no or mild spasticity.

The clinical assessments used in this study have been validated and are widely applied in stroke rehabilitation practice in Japan, and no specific permission was required for their use.

Written informed consent was obtained from all participants or their legal guardians, and the study was approved by the Ethics Committee of Okanami General Hospital, Mie, Japan (Approval Number: 0002).

Intervention

**Figure 1:**
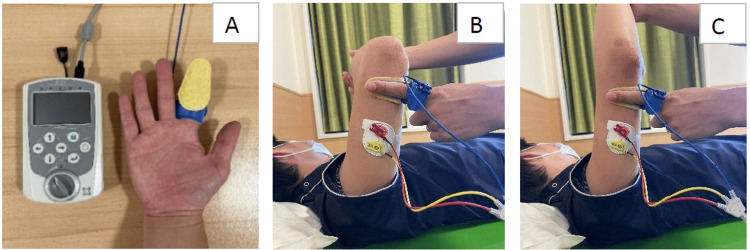
ES therapy using the IVES+ device with a FEE (A) The IVES+ device and FEE attached to a therapist’s hand. (B) Initiation of the elbow extension exercise: one electrode is placed proximally on the patient’s upper arm, and the patient attempts to extend the elbow from a flexed position. (C) The therapist delivers ES by contacting the target muscle with the FEE, synchronized with the patient’s motor intention, while simultaneously assisting the elbow extension movement. ES, electrical stimulation; FEE, finger-equipped electrode

ES therapy was administered using an IVES+ device (Model GD611; Oji Giken Co., Ltd., Tokyo, Japan) and FEE (Figure 1). One electrode was placed on the proximal portion of the patient’s target muscle, and the FEE was attached to the therapist’s index finger, enabling stimulation through direct contact. Participants were positioned supine, with ES applied to the target upper-limb muscles, while therapists simultaneously performed passive movements.

The therapist manually adjusted ES timing to match the participant’s movement intention, elicited through verbal cues. Participants were instructed to attempt voluntary movements during stimulation and focus on the stimulated limb. For example, during elbow extension, one electrode was placed on the proximal triceps brachii, the therapist verbally prompted the participant to “extend your elbow,” and the FEE was applied to the skin over the muscle to elicit contraction. In the presence of residual voluntary movement, the therapist provided minimal assistance to avoid interfering with the patient’s effort, whereas, in its absence, the therapist assisted the movement as needed.

Targeted movements included shoulder flexion and abduction, elbow extension, and wrist and finger extension. For shoulder flexion, the electrode was placed over the proximal portion of the anterior deltoid; for shoulder abduction, over the proximal portion of the middle deltoid; and, for wrist and finger extension, over the proximal portion of the extensor digitorum.

Based on a previous report [3], each movement was repeated 50-60 times (e.g., 20 stimulations × 3 sets). The number of stimulations per set was adjusted according to each participant’s fatigue level to prevent excessive loading. Stimulation frequency was set at 35 Hz, which induces clear muscle contractions while minimizing muscle fatigue [11]. Intensity was adjusted to the motor threshold that produced visible contractions without discomfort or pain.

Each occupational therapy session lasted 40-60 minutes, with approximately 30 minutes devoted to FEE-ES. The remaining time was used for other therapies, such as ADL training. Participants also received comprehensive multidisciplinary rehabilitation five days per week, including physical therapy (e.g., standing and gait training) and speech-language therapy (e.g., swallowing and voice training).

Outcome measures and data analysis

The primary outcome was upper-limb motor function, assessed using the FMA-UE. The upper-limb motor items of the SIAS, namely the Knee-Mouth and Finger Function tests, were also evaluated pre- and post-intervention to complement the assessment of motor function.

Furthermore, spasticity was assessed using the MAS to monitor changes in muscle tone, and any adverse events, such as skin irritation, were documented throughout the intervention period.

Outcome assessments were conducted by the treating occupational therapists, who also administered the FEE-ES intervention; thus, the assessors were not independent. Scores were compared between baseline (pre-intervention) and immediately after the one-month FEE-ES intervention (post-intervention).

The FMA-UE is a reliable and valid scale for assessing post-stroke upper-limb motor function, with a maximum score of 66 points. For acute stroke recovery, a minimal clinically important difference (MCID) of 10 points has been reported [12] and was used in this study to define clinically meaningful functional improvement. Statistical analysis was performed to compare pre- and post-intervention FMA-UE scores. Given the small sample size (n = 6), the Wilcoxon signed-rank exact test was applied. Effect sizes, $r = \frac{|Z|}{\sqrt{N}}$, were calculated to evaluate the magnitude of change. In addition, the Hodges-Lehmann estimator (median difference with 95% confidence interval (CI)) was calculated to provide a robust measure of central tendency and precision. The significance level was set at 5% (p < 0.05).

## Results

**Figure 2:**
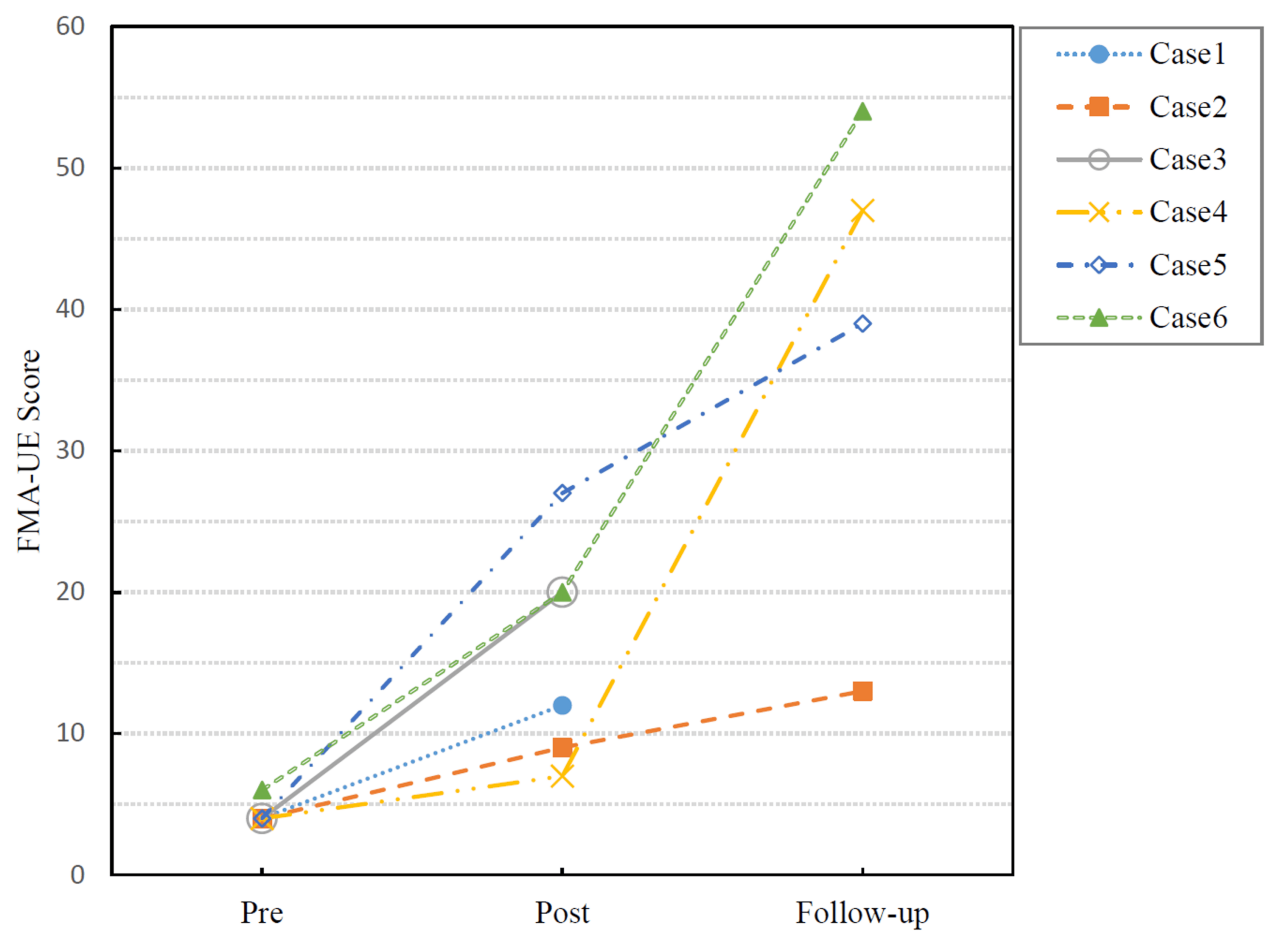
Changes in FMA-UE scores in six patients with acute stroke who underwent a one-month FEE-ES intervention Scores were assessed at pre-intervention (baseline), post-intervention (immediately after the one-month intervention), and follow-up (approximately one month after the intervention, i.e., two months after stroke onset). The trajectories of individual patients are presented. FMA-UE, Fugl-Meyer assessment for upper extremity; FEE-ES, finger-equipped electrode electrical stimulation

The FMA-UE scores for each participant are presented in Figure 2. All participants were assessed both pre- and post-intervention, and follow-up assessments were performed in four participants approximately one month after treatment completion. An improvement exceeding the MCID was observed in three of the six participants (Cases 3, 5, and 6). The remaining cases exhibited improvement but did not reach the MCID threshold. At follow-up, all four participants (Cases 2 and 4-6) demonstrated additional improvements, with Case 4 exhibiting a marked improvement.

The statistical results are summarized in Table 2. The median FMA-UE score increased from 4.0 (range: 4-6) at pre-intervention to 16.0 (range: 7-27) post-intervention. The Wilcoxon signed-rank exact test revealed a significant improvement (p = 0.031), with a large effect size (r = 0.86). The Hodges-Lehmann estimator indicated a median improvement of 11 points (95% CI: 3-23).

**Table 2:** Summary of pre- and post-intervention outcomes (FMA-UE, SIAS, and MAS) FMA-UE scores are presented for each case at pre- and post-intervention, along with the corresponding change values. The Wilcoxon signed-rank exact test revealed significant improvement in FMA-UE (p = 0.031), with a large effect size (r = 0.86). No significant increase in spasticity (MAS) or adverse events was observed during the intervention. FMA-UE, Fugl-Meyer assessment for upper extremity; MAS, modified Ashworth scale; SIAS-KM, stroke impairment assessment set-knee-mouth; SIAS-FF, stroke impairment assessment set-finger function

Case	FMA-UE			SIAS-KM		SIAS-FF		MAS	
	Pre	Post	Change (Post - Pre)	Pre	Post	Pre	Post	Pre	Post
1	4	12	+8	0	1	0	1	0	0
2	4	9	+5	0	1	0	0	0	0
3	4	20	+16	1	2	1	1	1	1
4	4	7	+3	0	1	0	0	0	0
5	4	27	+23	1	3	1	2	1	1
6	6	20	+14	0	3	1	3	0	0
Median	4	16	11	0	1.5	0.5	1	-	-
p-value	-	-	0.031	-	-	-	-	-	-
Effect size (r)	-	-	0.86	-	-	-	-	-	-

The SIAS motor scores for the upper limb also improved following the intervention. Specifically, the median SIAS-Knee-Mouth score increased from 0 to 1.5, and the median SIAS-Finger Function score increased from 0.5 to 1.0, indicating slight but consistent functional gains in proximal and distal movements.

Regarding safety outcomes, spasticity, which was assessed using the MAS, remained unchanged throughout the intervention period, with all participants scoring 0 or 1 both before and after FEE-ES. No participants experienced skin irritation or other adverse events during the intervention.

The follow-up data were already presented in Figure 2, but they were not included in the statistical analysis.

## Discussion

In the present study, we retrospectively examined six patients with acute-phase stroke and severe upper-limb paralysis, who underwent FEE-ES for approximately one month. Following intervention, the FMA-UE scores significantly improved compared with pre-intervention levels, with a large effect size (r = 0.86). Among the six participants, three (50%) achieved improvements exceeding the MCID of 10 points, indicating clinically meaningful functional gains in a subset of patients. Follow-up assessments were available for four participants approximately two months after stroke onset, revealing further score increases. However, as FEE-ES was discontinued after the initial intervention period, these improvements likely reflect ongoing recovery during standard rehabilitation and were not included in the main statistical analysis.

Only a few interventional studies have targeted patients with severe upper-limb paresis in the acute phase of stroke [13]. A recent meta-analysis of randomized controlled trials involving subacute stroke patients reported an average improvement of approximately 9.5 points in FMA-UE scores under standard care conditions after four weeks [14]. Although direct comparison is limited owing to differences in recovery phase and intervention protocols, the present study demonstrated a median improvement of 11 points, which appears slightly greater than that typically achieved with standard rehabilitation alone.

As ES enhances afferent input from muscles to the cerebral cortex and increases corticospinal tract excitability [15], combining it with voluntary movements further amplifies motor cortex excitability [16]. In a randomized controlled trial involving patients with severe upper-limb paralysis in the subacute phase, those receiving combined voluntary movement and ES achieved greater functional improvement than other groups [17], supporting the therapeutic importance of this approach. Notably, FEE-ES delivers ES through electrodes attached to the therapist’s fingers, requires no detectable muscle activity, and enables stimulation synchronized with the patient’s motor intent [3]. While most previous studies have focused on patients in the chronic phase, the acute phase is characterized by transient corticospinal tract impairment due to cerebral edema and penumbra, often resulting in severe paralysis. During this period, FEE-ES is particularly advantageous, as it provides synchronized stimulation even when voluntary movement is difficult. Furthermore, the greatest recovery after stroke typically occurs early, when neural plasticity is the highest [18,19]. Delivering appropriately timed sensory inputs and motor outputs during this window may promote corticospinal tract reorganization and neural network reconstruction. In patients with acute-phase stroke and severe upper-limb paralysis, a study comparing conventional intervention plus NMES with conventional intervention alone demonstrated significantly greater functional improvement in the NMES group than in the other groups [20]. Unlike EMG-triggered or periodic NMES, FEE-ES synchronizes stimulation with motor intent, even in the absence of measurable voluntary movement, enhancing its applicability in the acute phase.

In the present study, although significant improvements were observed following FEE-ES, not all patients exhibited comparable gains. These variations may be related to stroke severity, lesion location, or individual recovery potential. The MCID results indicate that FEE-ES may be particularly beneficial for a subset of patients; however, further research is required to identify predictors of response. Notably, no increase in spasticity or skin complications occurred, and all participants completed the one-month program without adverse events, indicating that FEE-ES is feasible and well-tolerated in acute, severe cases.

This study has certain limitations. First, the sample size was small (n = 6), limiting statistical power and generalizability. Additionally, the gender distribution was unbalanced, which may further limit the generalizability of the findings. Second, the study lacked a control group, making it difficult to differentiate the effects of FEE-ES from natural recovery or concurrent rehabilitation. Third, outcome measures were restricted to the FMA-UE, and multidimensional assessments, such as ADLs and QOL, were not performed. Fourth, follow-up data were obtained after FEE-ES discontinuation, during which participants received standard multidisciplinary rehabilitation only. Therefore, these findings cannot be attributed to FEE-ES and were treated as supplementary information. Fifth, the outcome assessments were conducted by the treating therapists who provided the intervention, which may have introduced observer bias. Finally, although FEE-ES was designed to synchronize stimulation with the patient’s motor intent, objective measures, such as surface EMG, were not included to confirm synchronization accuracy. Future studies incorporating neurophysiological measurements are warranted to validate this mechanism.

Moreover, identifying clinical predictors of favorable response, such as lesion location or baseline impairment severity, is important for optimizing patient selection and maximizing therapeutic efficacy.

## Conclusions

This retrospective case series demonstrated that FEE-ES during the acute phase of stroke is feasible, well-tolerated, and associated with significant improvements in upper-limb motor function in patients with severe paralysis. Some individuals demonstrated continued functional gains beyond the acute stage, suggesting that early synchronization of motor intent with ES may promote long-term recovery. Although preliminary, these findings support the potential of FEE-ES as an adjunct rehabilitation strategy, and highlight the need for controlled studies to confirm efficacy and optimize protocols.
